# On The Quotient of a Centralized and a Non-centralized Complex Gaussian Random Variable

**DOI:** 10.6028/jres.125.030

**Published:** 2020-09-23

**Authors:** Dazhen Gu

**Affiliations:** 1 National Institute of Standards and Technology, Boulder, CO 80305, USA

**Keywords:** complex random variables, Rayleigh distribution, Rice distribution

## Abstract

A detailed investigation of the quotient of two independent complex random variables is presented. The numerator has a zero mean, and the denominator has a non-zero mean. A normalization step is taken prior to the theoretical developments in order to simplify the formulation. Next, an indirect approach is taken to derive the statistics of the modulus and phase angle of the quotient. That in turn enables a straightforward extension of the statistical results to real and imaginary parts. After the normalization procedure, the probability density function of the quotient is found as a function of only the mean of the random variable that corresponds to the denominator term. Asymptotic analysis shows that the quotient closely resembles a normally-distributed complex random variable as the mean becomes large. In addition, the first and second moments, as well as the approximate of the second moment of the clipped random variable, are derived, which are closely related to practical applications in complex-signal processing such as microwave metrology of scattering-parameters. Tolerance intervals associated with the ratio of complex random variables are presented.

## 1 Introduction

Complex data are often encountered in communication signals for convenience in modeling and processing. Although communication signals are originally all real valued, the signals produced and detected by communication and measurement instrumentation can be practically split into the inphase and quadrature components, which form the real and imaginary parts of a complex representation of the signal. Additionally, complex signals in the time domain can be easily processed with complex Fourier transformations to acquire their representation in the frequency domain and vice versa. In the frequency domain, the inphase and quadrature components can be processed to extract the upper- and lower-side bands of the signals. This, in turn, reproduces the entire envelope of the signal in a band of interest.

All the manipulation in the complex domain is also applicable to naturally random signals, such as noise. Due to its ubiquity, noise is present in all stages of signal processing, no matter how the signals are produced, transmitted and detected. Furthermore, band-limited noise is commonly modeled by a complex random variable (RV) with independent and identically distributed (i.i.d.) real and imaginary parts. As a result, the statistics of complex Gaussian RVs find utility in many applications of signal processing. Schoenecker and Luginbuhl derived the characteristic function for the product of two Gaussian RVs [1] Astely and Ottersten studied the ratio of zero-mean and non-zero-mean complex RVs to investigate the angle-of-arrival due to local scatterers of wireless receivers in [2]. Baxley *et al* derived the joint probability density function (PDF) between two general zero-mean complex Gaussian RVs and used that for calculating the symbol error rates of signals in fading channels [3]. More recently, Wu and Hughes dealt with a complex Gaussian ratio for finding the maximum likelihood estimator of the joint channel and antenna impedance [4]. Wu also expanded the previous result [4] to show high-order moments of the magnitude of the quotient [5]. On other fronts, Nadimi *et al.* presented derivations of a closed-form expression of the ratio of two independent non-zero mean complex Gaussian RVs [6]. Extending Nadimi's work, Nadarajah and Kwong derived more manageable expressions for improved computation efficiency [7]. Most recently, Li and He provided the derivation of the PDF and the first moment for the most generic complex Gaussian ratio [8].

In an ongoing effort [9], a model is under development for studying the influence of noise on scattering-parameter measurements performed by vector network analyzers (VNAs). Scattering parameters are critical microwave parameters that characterize attenuation and phase-delay ascribed to a device under test (DUT). During the measurement process, noise is added by various signal-generation and signal-detection components inside the VNA, as well as passive or active electronics in the DUT. The preliminary study focus on the variability of noisy scattering parameters corresponding to the ratio of complex RVs. This paper aims to provide a detailed development of th⁓ry for many statistical results quoted in Ref. [9], although we expect to extend this to other relevant applications. A summary of the literature comparison is listed in Table 1. New results in this paper include a new approach for deriving PDFs of the complex Gaussian quotient, asymptotic analysis of the distribution function and moments of the quotient at high and low limits, and tolerance intervals for quantifying the variability of the measured ratios of complex RVs.

**Table 1:** Literature comparison.

Contributions	[2]	[3]	[4, 5]	[6, 7]	[8]	This paper
Complex Gaussian quotient	* $\frac{CN(0,\sigma_{X}^{2})}{CN(\mu_{y},\sigma_{y}^{2})}$ *	$\frac{CN(0,\sigma_{X}^{2})}{CN(0,\sigma_{y}^{2})}$	* $\frac{CN(\mu_{x},\sigma_{X}^{2})}{CN(\mu_{y},\sigma_{y}^{2})}$ *	* $\frac{CN(\mu_{x},\sigma_{X}^{2})}{CN(\mu_{y},\sigma_{y}^{2})}$ *	$\frac{CN(\mu_{x},\sigma_{X}^{2})}{CN(\mu_{y},\sigma_{y}^{2})}$	$\frac{CN(0,\sigma_{X}^{2})}{CN(\mu_{y},\sigma_{y}^{2})}$
Correlations		✓	✓		✓	
Joint PDF		✓		✓	✓	✓
PDF of real part	✓					✓
Moments	_1_st		_1_st_, 2_nd _&_ *_k_*th		_1_st	_1_st _& 2_nd
Asymptotic analysis						✓
Tolerance intervals						✓

## 2 Statement of Problem and Normalization of RVs

The goal is to study the statistical properties of *Z*, which is the ratio of two independent complex Gaussian RVs, *X* and *Y* . The numerator *X* has the distribution *CN(*0, $\sigma_{x}^{2}$), while the denominator *Y* has the distribution *CN* (*μ_y_,* $\sigma_{y}^{2}$), where the real and imaginary parts are i.i.d. RVs. The complex normal notation *CN* (·, ·) implies the following information. The means of *X* and *Y* are 0 and *μ_y_* respectively. The variance of the real part and the imaginary part of *X* is (*$X_{r}^{2}$*) = (*$X_{i}^{2}$*) =*$\sigma_{x}^{2}/2$*. The same identity is applied to *Y* ; namely ($Y_{r}^{2}$) - (*Y_r_*)^2^ = ($Y_{i}^{2}$) - (*Y_i_*)^2^ =$\sigma_{y}^{2}/2$. In addition, *X_r_*, *X_i_*, *Y_r_* and *Y_i_* are all normally distributed and they're mutually independent. Here, the subscripts *r* and *i* indicate, respectively, the real and imaginary parts of the complex number.

In general, *μ_y_* can be a non-zero complex number given by *μ_y__r_* + ℐ*μ_y__i_* , where ℐ =$\sqrt{-1}$ is the imaginary unit. Although it's possible to proceed as is, the formalism soon becomes difficult to handle. Instead, we first apply normalization on the two RVs for ease of developments.

To normalize the complex RVs, a linear transformation is performed as follows,


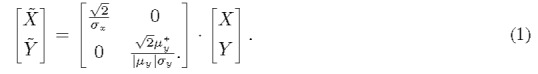


Here $\cdot^{*}$ indicates the complex conjugation operation and |⋅| is the modulus of a complex number.

Now $\tilde{X}$ has the distribution 𝒞⁢𝒩⁢(0, 2) and $\tilde{Y}$ has the distribution $𝒞\,𝒩\,\left(\sqrt{2\,S},2\right)$, where $S={\left|\mu_{y}\right|}^{2}/\sigma_{y}^{2}$. Note that the mean of Y is transformed to a positive real quantity as a function of S ^1^1In practical applications, S is the signal-to-noise ratio (SNR).. As a result, the following PDFs of independent RVs are readily obtained.


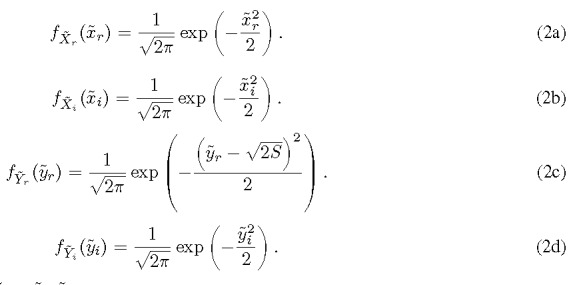


The ratio of the new RVs, $\tilde{Z}=\tilde{X}/\tilde{Y}$, is related to the original Z by


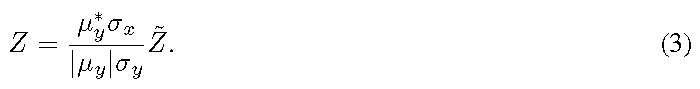


In general, the statistical properties of *Z* can be easily deduced from those of *$\overset{⁓}{Z}$*. For example, the joint PDF of the real and imaginary parts is related with


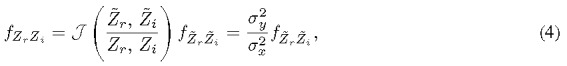


where 𝒥⁢(⋅) is the determinant of the Jacobian. The mean and the covariance of $Z_{r}$ and $Z_{i}$ can be obtained from those of ${\tilde{Z}}_{r}$ and ${\tilde{Z}}_{i}$ with the linear operations


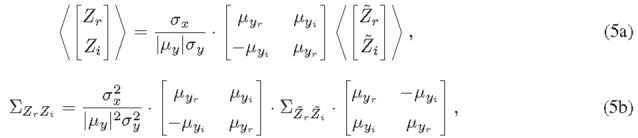


where the angle bracket is the statistical ensemble average. As will be shown later in Sec. 3.4, ${\overset{⁓}{Z}}_{r}$ and ${\overset{⁓}{Z}}_{i}$ are identically distributed and uncorrelated. These characteristics of ${\overset{⁓}{Z}}_{r}$ and ${\overset{⁓}{Z}}_{i}$, in combination with Eqs. (4), (5a) and (5b), make $Z_{r}$and $Z_{i}$ also identically distributed and uncorrelated. Note that ${\overset{⁓}{Z}}_{r}$ and *${\overset{⁓}{Z}}_{i}$*are shown to be dependent in Sec. 3.4.1 since their joint PDF cannot be factorized into the product of the individual PDFs.

In practice, the independence between *X* and *Y* has been validated experimentally [9]. Additionally, the values of *σ_x_*, *μ_y_* and *σ_y_* can be obtained from many realizations of the complex RVs *X* and *Y* by performing repeated measurements of noise in a measurement system. Next, the value of *S* can be estimated by following the normalization procedure outlined above.

In the following, we focus our efforts on dealing with RVs $\overset{⁓}{X},\overset{⁓}{Y}$, and the resultant $\overset{⁓}{Z}$. As will be readily seen, this manipulation greatly simplifies the formulation. We also remove the $\overset{⁓}{\cdot }$ on various RVs to simplify the notation in what follows. Along with the derivation of the statistics of the complex Gaussian quotient, general properties of the normally distributed complex RVs are used extensively. Some of these basic properties are discussed in Appendix B.

## 3 Distribution of Magnitude and Phase of Quotient

After normalization, the quotient is simply an RV with a distribution of CN(0, 2) divided by another RV with a distribution of *CN*($\sqrt{2S}$, 2). Evidently, direct derivation of the PDFs of the real and imaginary parts of *Z* presents a formidable challenge since either of them is a quotient consisting of nonlinear functions of four RVs, *X_r_*, *X_i_*, *Y_r_*, and *Y_i_*. In Ref. [2], Astely derived the distribution function by calculating the conditional probability function of *f_Z__r_*_|_*_Y_* . Here, we start by finding the PDF of the modulus (*R_z_*) and the phase angle (Θ*_z_*) of *Z* in polar coordinates. Next, *f_Z__r_* can be calculated from the marginal distribution of the joint PDF *f_Z__r__Z__i_* , which is directly obtainable from *f_R__z_*_Θ_*_z_* . The knowledge of the distribution function and the moments of *R_z_* finds its direct use in validating the model with simulation and experimental data, as the radius of the scattered complex data can be conveniently acquired from many realizations.

## 3.1 Distribution of R_x_

The RV *R_z_* is simply the quotient of a Rayleigh RV (*R_x_*) [10] divided by a Rician RV (*R_y_*) [11]. Since RVs *X* and *Y* are independent, their moduli are also independent. To acquire *f_R__x_* , we can first find the cumulative distribution function (CDF) *F_R__x_* (*r_z_*) or equivalently the probability of *R_x_* ≤ *r_z_R_y_*.


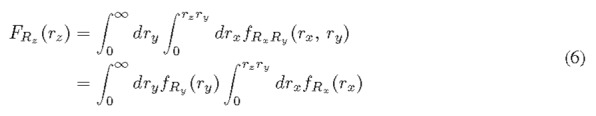


Applying the derivative on Eq. (6) with respect to *r_x_* results in the PDF:


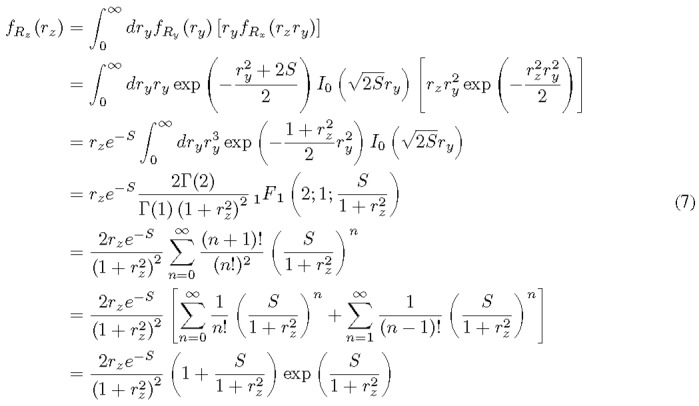


Here, the standard formulae of the Rice distribution and the Rayleigh distribution are used in the second step. *I*_0_(·) is the modified Bessel function of the first kind at zeroth order. Γ(·) is the gamma function. _1_*F*_1_(·;·;·) is Kummer's hyperg⁓metric function [12, Chapter 13]. The integration result on the fourth line of Eq. (7) is due to Hankel [13, Eq. (13.3.2)]. Note that the PDF in Eq. (7) only applies to *r_z_* ≥ 0 since the modulus is a non-negative quantity.

The moments of *R_z_* may be of practical interests in many applications. We study the first and the second moments in the rest of this section.

## 3.1.1 First Moment of R_z_

We start with the definition of the first moment and derive its expression as follows.


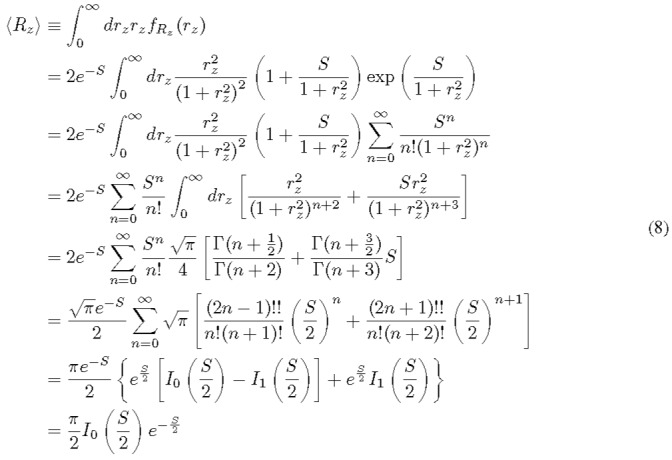


Here, *I*_1_(·) is the modified Bessel function of the first kind at first order. This result is consistent with that in Ref. [2], although the authors did not recognize that the specific confluent hyperg⁓metric function can be simplified as the result in Eq. (8). By use of the expansion of the modified Bessel function and the exponential function at small and large *S* limits, we can further show


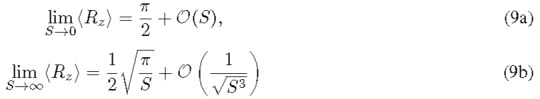


The symbol 𝒪⁢(⋅) means on the order of. The following expansions are used [12, Eq. (10.25.2) and Eq. (10.40.1)] for deriving Eq. (9).


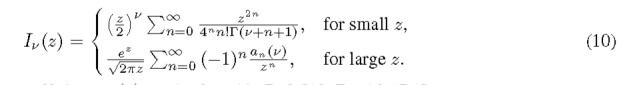


Here the expression of the coefficient *a_n_*(*v*) can be found in Ref. [12, Eq. 10.17.1].

## 3.1.2 Second Moment of R_z_

Following the similar procedure, we can show the second moment as follows.


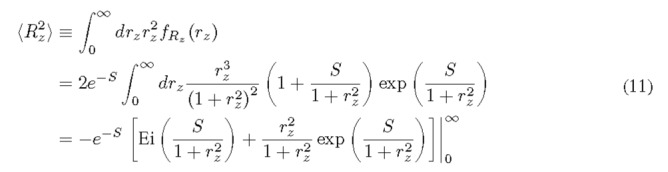


Here, Ei(·) is the exponential integral function. Equation (11) is divergent for any finite *S* because of the singularity of Ei(0). The divergence of the moments was also recognixed by several authors in published literature [2, 4, 5, 14]. For a clipped version of *R_x_*, where we set the maximum value of *r_x_* (namely *r*_max_) to a finite value, we provide an accurate approximation to the second moment of the clipped version of *R_x_* for cases studied. The second moment of the clipped RV can be expressed by


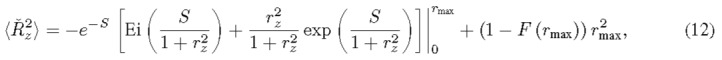



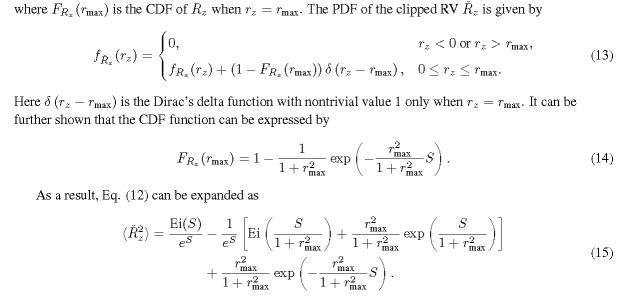


The enforced finite bound is reasonable in practical realixation because there is an upper detection limit (*r*_max_) associated with the measurement devices (detectors or instruments). Any signals above a certain level will cause saturation and the detected value is clipped. From a statistical standpoint, experimental results correspond to a clipped version of the RV *R_x_*. Although it's possible to use Eq. (15) directly to estimate the second moment, it's not uncommon that precise knowledge of *r*_max_ is unavailable. Since several instruments or sensors are usually involved in measurements, determining the stage at which the saturation actually occurs is not straightforward. As an approximate approach, we rely on the first term in Eq. (15) that is independent of *r*_max_.


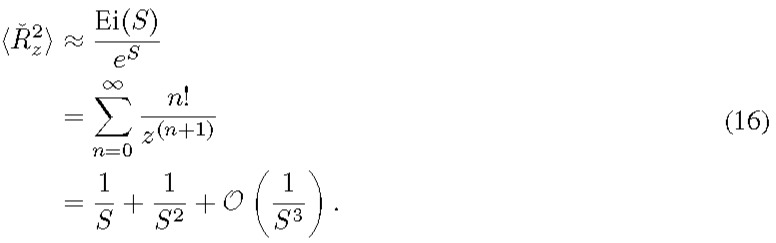


Here, the series expansion can be inferred from the identity of the exponential integral function as follows [12, Eq. (6.12.2)]


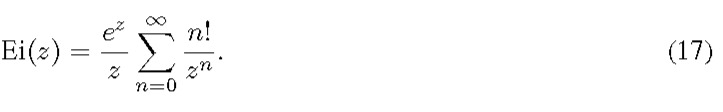


**Fig. 1:**
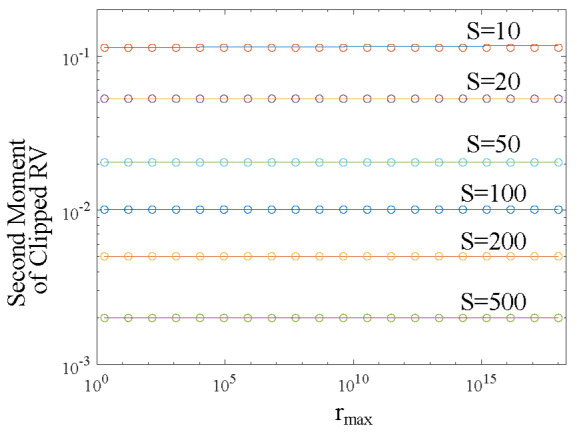
Comparison between calculated Eq. (15) (lines) and approximate Eq. (16) (circles) results of the second moment of the clipped RV *R*^E^*x* . The approximate expression used was Ei(*S*)*/e^S^*. The minimum *F* (*r*max) in this study was 0.99993290747442 for *S* = 10 and *r*max ^= 2.^

For a large argument, it is unwieldy to evaluate the exponential function and the exponential integral function through a numerical computation tool. Instead, evaluation of the series to a certain order is more convenient. We ran comparison between Eq. (15) and Eq. (16) by numerical calculation. An excellent agreement was reached with the relative error less than 0.7% for *S* ≥ 10 and *r*_max_ ⋳ [2, 10^17^], as shown in Fig. 1.

As noted earlier, the second moment of the unclipped *R_x_* is unbounded. Hence, the standard deviation of the unclipped *R_x_* is not well-defined. However, we can quantify the variability of *R_x_* by stating the upper and lower endpoints of a tolerance interval even though the standard deviation of the unclipped *R_x_* is not well-defined.

## 3.2 **PDF of** Θ*_y_*

In order to obtain the PDF of Θ*_Z_*, it seems necessary to know the PDFs of both Θ*_x_* and Θ*_y_*, although it will soon be uncovered that the knowledge of PDF of Θ*_y_* is not required. Nevertheless, we investigated the PDF of Θ*_y_* for the sake of completeness and potential needs for practical applications.

**Fig. 2:**
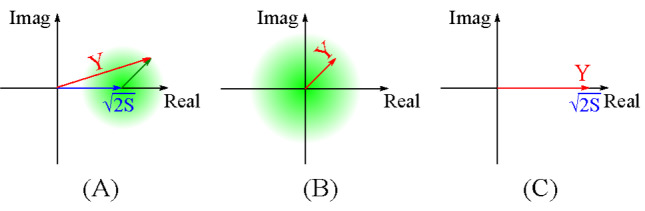
(A) Graphical illustration of vector *Y*, the sum of a fixed real vector and a random vector of unit real and imaginary RMS length. (B) Low SNR limit. (C) High SNR limit.

The RV *Y* represents a deterministic real number ($\sqrt{2S}$) perturbed by the noise as shown in Fig. 2(A). Both real and imaginary parts of the noisy vector have a unit root-mean-square (RMS) length. Overall, RV *Y* follows the distribution CN($\sqrt{2S}$, 2). Rice provided the distribution of its modulus *R_y_*, which later became the well-known Rice (or Nakagami-n) distribution [11]. Although various pertinent RVs, such as the time derivatives ${\overset{\cdot }{R}}_{y}$ and Θ^·^ *_y_* , were extensively studied, the statistics of the phase angle Θ*_y_* were not available. This missing piece is furnished in this section.

To determine the PDF of Θ*_y_*, we start with calculating the marginal probability from the joint PDF of *R_y_* and Θ*_y_*.


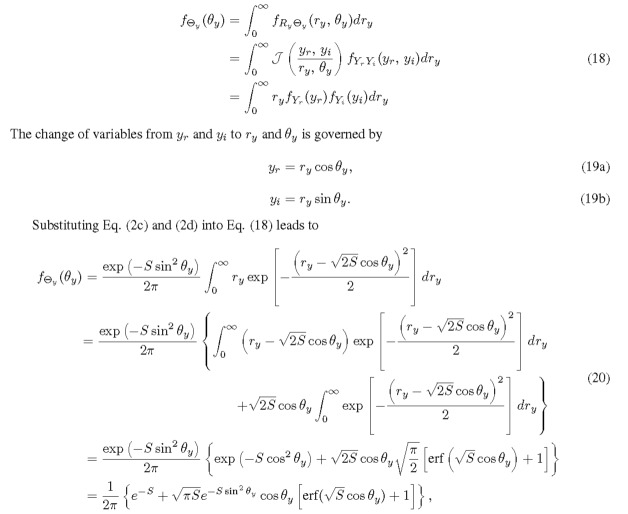


where erf is the error function. The CDF convergence of *f*_Θ_*_y_* is presented in Appendix A.

It is of practical interest to study *f*_Θ_*_y_* at high and low limits of *S* (SNR). For low SNRs, the distribution


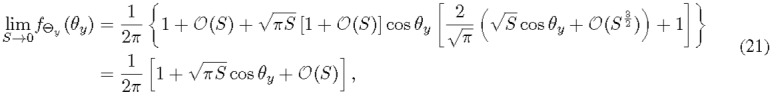


where the expansions of the exponential function and the error function are used. Not surprisingly, the PDF in Eq. (21) approaches the uniform distribution. The graphical illustration is shown in Fig. 2(B). When the noise dominates, i.e. where the SNR is low, random vector *Y* reduces to the noise vector and hence its angle distribution follows the uniform distribution.

For high SNRs, it is easy to show by use of the error function limits at ±∞


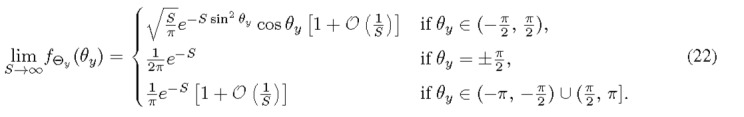


Equivalently, the probability only has significant values near *ϴ_y_* = 0. This is as expected, since the vector *Y*

essentially lies on the real axis with negligible perturbation when the SNR is high.

## 3.3 **PDF of** Θ*_z_*

We now can proceed to find the PDF of $\Theta_{z}$. Although the distribution can be intuitively postulated as a uniform distribution, we verify its validity with the following derivation. Since its principal value must be limited in the interval of (−π,π], $\Theta_{z}$ is not just the difference between $\Theta_{x}$ and $\Theta_{y}$. Instead, it can be expressed in three cases as follows.


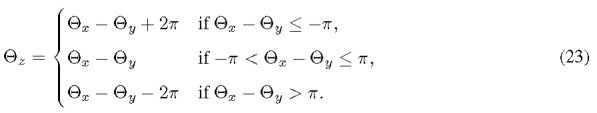


**Fig. 3:**
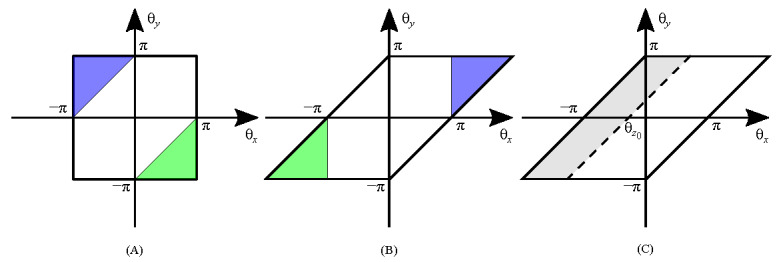
Transformation of the two-dimensional area from the original (A) to (B) according to the periodicity in 2*π*. The shaded region in (C) indicates the integration domain for calculating the probability.

To obtain its PDF, we start with the CDF of Θ*_z_* and then perform a derivative. Equivalently, the probability of Θ*_z_* for *ϴ_z_* ∈ (-*π, ϴ_z_*_0_] is needed. Referring to Fig. 3, the squared region of *ϴ_x_* and *ϴ_y_* is transformed to a parallelogram according to Eq. (23). The area in Fig. 3(C) for calculating the probability can be expressed as


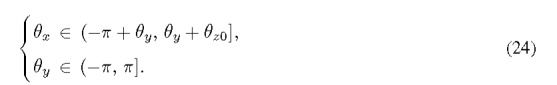


Therefore the probability is given by


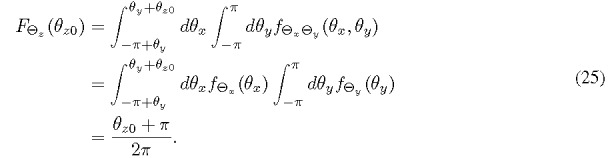


Here, we use the fact that Θ*_x_* is uniformly distributed and its PDF is invariant with the periodicity of 2*π*. Equation (25) evidently shows that Θ*_z_* is also uniformly distributed. Note that no additional constraints on .*f*_Θ_*_y_* need to be imposed for Eq. (25) to hold except that Θ*_y_* is independent from Θ*_x_*. As a consequence, we can extend our finding to a more general theorem: *A linear combination of independent phase-angle RVs is uniformly distributed as long as at least one of them is uniformly*

## 3.4 Statistics of *Z_r_* and *Z_i_*

With *f_R__z_* and *f*_Θ_*_z_* at our disposal, we are ready to determine the statistics of *Z_r_* and *Z_i_*. Prior to tackling the joint PDF of *Z_r_* and *Z_i_*, we need to clarify the independence between *R_z_* and Θ*_z_*. Their independence relies on the following facts.

· *X_r_*, *X_i_*, *Y_r_* and *Y_i_* are mutually independent,

· Their polar representations *R_x_*, *R_y_*, Θ*_x_* and Θ*_y_* are also mutually independent,

· *R_z_* is a function of *R_x_* and *R_y_* only,

· Θ*_z_* is a function of Θ*_x_* and Θ*_y_* only.

## 3.4.1 PDF, Moments and Covariance

Because of their independence, the joint PDF of *R_z_* and Θ*_z_* is simply the product of *f_R__z_* and *f*_Θ_*_z_* . That in turn allows us to deduce the joint PDF of *Z_r_* and *Z_i_* as


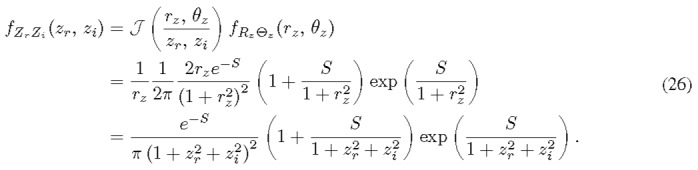


Here, the Jacobian determinant can be found from the transformation from the Cartesian coordinate system to the polar coordinate system. The joint PDF of *R_z_* and Θ*_z_* is the product of their individual PDFs (Eq. (7) and the derivative of Eq. (25)), since they are independent.

The individual PDFs can be calculated from the marginal distribution of Eq. (26). Because of the symmetry, it is straightforward to see that the RVs *Z_r_* and *Z_i_* distribute in the same way. We proceed to calculate *f_Z_* (*z_r_*) with a change of variable *I* % 1 + *z*^2^ for a short-handed notation.


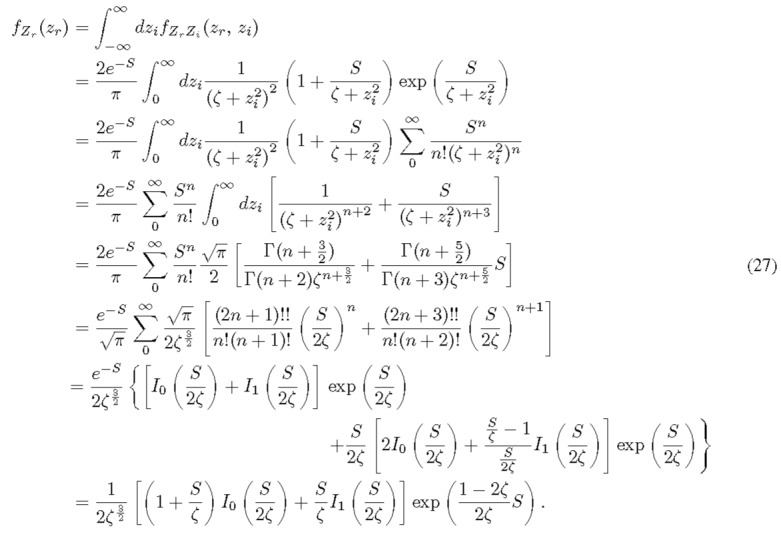


Not surprisingly, *f_Z__r_* (or *f_Z__i_* ) is an even function of *z_r_*. Therefore, the means of both *Z_r_* and *Z_i_* are zero. The second moment of *Z_r_* (also its variance in this case) is half of $\langle R_{z}^{2}\rangle$, since $\langle Z_{r}^{2}\rangle$ and $\langle Z_{i}^{2}\rangle$ are identical.

As a consequence, $\langle Z_{r}^{2}\rangle$ is generally divergent except at large *S*. From Eq. (16), we have


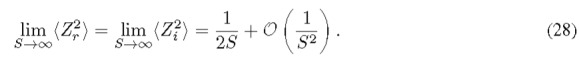


Equivalently, the standard deviation, denoted by $\sigma_{Z_{r}}$, at high S limit is $1/\sqrt{2\,S}$ or $\sqrt{2/\pi }\,\left\langle R_{z}\right\rangle$.

$Z_{r}$ and $Z_{i}$ are uncorrelated since their covariance is


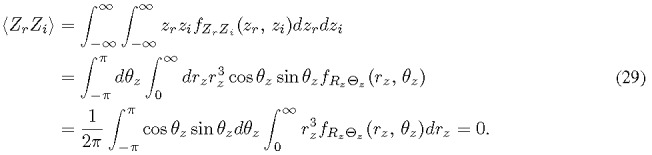


It may be tempting to conclude *Z_r_* and *Z_i_* are independent. However, RVs *Z_r_* and *Z_i_* are actually dependent since their joint PDF cannot be factored into the product of *f_Z__r_* and *f_Z__i_* as shown in Eq. (26).

## 3.4.2 PDF at High SNR

As *S* becomes large, it is expected that *Z* will closely resemble a centralized complex RV with distribution CN(0, 2*/S*). It is worth checking to see the asymptote of the distribution function coincides with the prediction. We start by the expansion of the modified Bessel function with a large argument, as shown in Eq. (10).


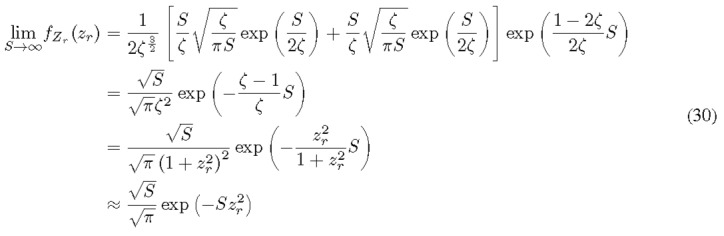


**Fig. 4:**
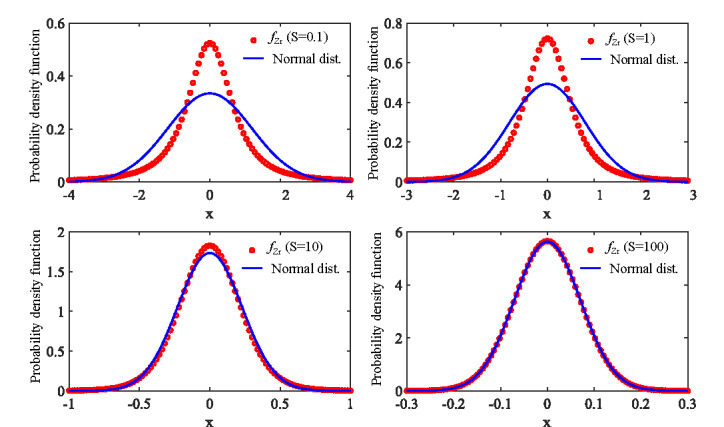
PDFs of the real part of the complex Gaussian quotient as a function of *S* from Eq. (27) in comparison to the normal distribution approximation shown in Eq. (30).

Indeed, this is equivalent to a zero-mean normally distributed RV with a variance of 1*/*(2*S*). The approximation made at the last step is also due to the large value of *S*. The probability density drops exponentially as the magnitude of *z_r_* grows. In other words, the PDF only holds significance when |*z_r_*| A" 1. Consequently, the limit of small |*z_r_*| can be applied to reach the final distribution function in Eq. (30). The normal approximation to the distribution of the RV of interest from Eq. (27) is asymptotically valid as S increases, shown in Fig. 4.

## 3.4.3 Tolerance Interval

For metrological applications, it is valuable to study the tolerance interval of RVs [15, 16]. The uncertainty budget for physical parameters of interest will depend on the variability of measured quantities as well as uncertainties associated with calibration artifacts and other instrumental effects. Hence, tolerance intervals should provide a guidance on the relative contributions of particular sources of measurement variability to the overall uncertainty budget. A metrologist can make a judgment call whether the noise effect is worth consideration and large numbers of repeated measurements need to be conducted to extract the probability distribution of the RVs.

For this complex quotient RV, the tolerance interval of its real (or imaginary) part can be calculated from the *q*th quantile associated with the model PDF shown in Eq. (27). In essence, we need to solve the bounds for a given tolerance (1 ^' *q*) by determining the interval FZr-1q/2,FZr-1S,1^'q/2. The CDF of *Z_r_* is simply


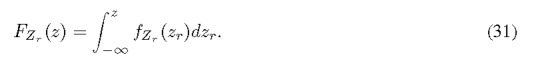


In practical applications, *S* corresponds to the SNR of the excitation signal. The value of *S* can be estimated from the experiments, so that for a given tolerance value, we will be able to invert the CDF function to obtain the bounds for *Z_r_* (or *Z_i_*) under a specified SNR condition.

**Fig. 5:**
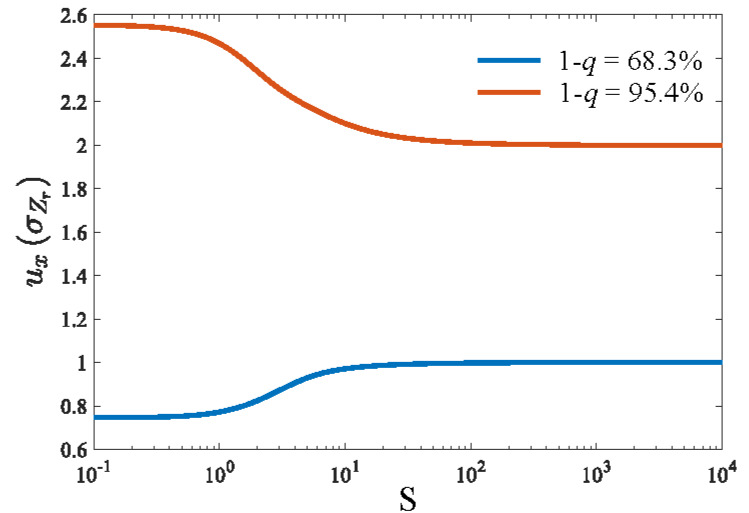
Upper endpoints for 68.3% and 95.4% tolerance intervals. The RVs are modeled according to Eq. (27). The endpoints are normalized by $\sigma_{Z_{r}}=\sqrt{\frac{2}{\pi }}\langle R_{z}\rangle$

For normally distributed RVs, values of particular interest are q=1^'erf1/2≈31.7% and 1 - erf$\left(\sqrt{2}\right)$ ≈ 4.6%. Numerical results for the these two tolerance interval are shown in Fig. 5. Evidently, at large *S*, [-*I_Z__r_, I_Z__r_* ] and [-2*I_Z__r_,* 2*I_Z__r_* ] result in 68.3% and 95.4% tolerance intervals, respectively. Once again, this behavior is consistent with the normal distribution derived in Sec. 3.4.2. However, these bounds vary as a function of *S* since the distribution deviates from the normal distribution as *S* decreases.

For small *S* values, [-0.75*σ_Z__r_,* 0.75*σ_Z__r_* ] corresponds to the 68.3% tolerance interval while

[-2.55*σ_Z__r_,* 2.55*σ_Z__r_* ] corresponds to the 95.4% tolerance interval.

**Fig. 6:**
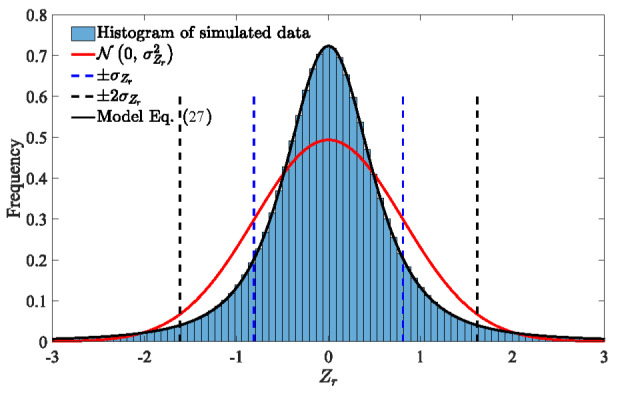
Histogram of simulation compared to standard normal distribution *N* (0*,*$\sigma_{Z_{r}}^{2}$). Bounds of intervals of [*-σ, σ*] and [*-*2*σ*, 2*σ*] are shown with the dashed lines. Note that the simulation results were obtained with *S* = 1. The model PDF of Eq. (27) (black line) and the approximate normal distribution from Eq. (30) (red line) are also plotted for comparison.

It may seem puzzling that the two tolerance intervals do not vary the same way. The 68.3% tolerance interval, shrinks as *S* decreases, while the 95.4% tolerance interval, expands. This is solely due to the PDF of *Z_r_*. An example for *S* = 1 is shown in Fig. 6, where the histogram normalized to the PDF of the simulated data is compared to the standard normal distribution. Although the PDF of *Z_r_* is more concentrated in the central region around *Z_r_* = 0, the probability density of *Z_r_* reduces more rapidly than that of a normally distributed RV when *Z_r_* grows bigger. As a result, a broader interval is required to reach a high tolerance, such as 95.4%.

## 4 Conclusion

The quotient of a zero-mean complex Gaussian RV and a non-zero-mean complex Gaussian RV was investigated in this paper. The PDF of the real (or the imaginary) part was derived by first obtaining the joint PDF of the modulus and the phase of the complex Gaussian ratio, followed by marginalizing the joint PDF. Asymptotic analysis showed that such a complex Gaussian quotient approaches a normally distributed complex RV under the condition when the mean of the denominator is much larger than the variance of the denominator. In addition, the tolerance interval of the complex Gaussian quotient was derived by numerically calculating the related quantile of the distribution.

The results in this paper have many applications in microwave scattering-parameter measurements. The first moment of the modulus of the complex Gaussian quotient corresponds to the radius of the scattering-parameter "cloud" obtained from repeated measurements. The radius of such a "cloud" can be compared to the theoretical predication in Eq. (9) for noise-modeling validation. In addition, the second moment of the modulus can be used in isolating the noise contributions in power-related measurements in microwave reverberation chambers. Noise contributions are embedded together with random variations introduced by paddle rotations in reverberation chambers. Paddle rotation effects are determined in the presence of noise based in part on the model shown in Eq. (16). Furthermore, the tolerance intervals related to the random variations can help metrologists to assess the impact of noise on scattering-parameter measurements. There are various uncertainty sources in the VNA measurements. The variability of the SNR-dependent RV can be determined, so that a fair comparison can be drawn between noise-induced variations and measurement uncertainties, especially when SNR is low.

## 5 Appendix A

At a glance, the PDF developed in Eq. (20) does not seem to converge to 1 over the integration range of

[-*π, π*]. We provide its convergence proof as follows.

The rationals of the critical steps are listed below.

• Step *a*: The integrand is an even function and is also symmetrical about ${ϴ}_{y}=\frac{\pi }{2}$
• Step *b*: There is a change of the variables from *Θ_y_* to *t* by *$t=\sqrt{S}$* cos *ϴ_y_*.
• Step *c*: The integration can be referred to Ref. [18, Eq. (4.3.19)]. This result can be reproduced by recursive integral by parts that leads to an infinite series equivalent to the exponential function. We omit the derivation details here.

## 6 Appendix B

In this appendix, properties of complex RVs representing noise are presented. The real and imaginary parts of the RVs represent, respectively, the inphase and quadrature components of the noise signal at the frequency of interest. In common scenarios of communication electronics, the bandwidth of the signal (or noise) channel is very small compared to the carrier frequency *f*_0_. Within this narrow band, the power spectral density of the noise can be considered symmetrical about the center of the frequency band. As such, the real and the imaginary parts of the RVs are uncorrelated. It is also well known that uncorrelated Gaussian RVs are independent. Additionally, the variances of both the real and the imaginary parts of the complex RV are equal to the sum of the noise power in the upper- and lower-side bands of the noise.

Consequently, the complex RVs modeling band-limited noise can be constructed with i.i.d. RVs as follows

Both the real part and the imaginary part are zero-mean and normally distributed with an identical variance, $X_{r},X_{i}∼𝒩\,\left(0,\sigma_{x}^{2}/2\right)$. The joint PDF of $X_{r}$ and $X_{i}$ takes the form

For this kind of complex RV, its statistical properties are invariant of phase rotation, i.e. circular symmetry. To see this property, we apply an arbitrary phase shift α on X, so that the new phase-shifted complex RV is $\overset{˘}{X}=X\,e^{ȷ\,\alpha }$ and the new real and imaginary parts are

where det is the determinant of a matrix. The identities shown above prove that the statistical properties of *X*^E~^ and *Y*^E~^ remain unchanged and the circular symmetry property is rotationally invariant.

In many practical interests, it is helpful to know the distribution of the magnitude of the complex RV. It is essentially a chi-square distribution with two degrees of freedom, also known as the Rayleigh distribution [10], expressed by ${ƒ}_{R_{y}}=\frac{r_{x}}{\sigma_{x}^{2}}\exp\left(-\frac{r_{x}^{2}}{2\sigma_{x}^{2}}\right).$

When noise is presented in combination with a deterministic signal, the model becomes a non-zero mean complex RV: *Y* = *A_y_* + *X*. If *A_y_* is a complex number, a phase shift operation (*I* = -Arg(*A_y_*)) can be applied on *Y* to reach a new non-zero mean complex RV: $\overset{͝}{Y}=\left|\mu_{y}\right|+Xe^{Ϳ\text{Arg}(\mu_{y})}$. Once again, the statistical property of the phase-rotated *X* remains unchanged due to its circular symmetry. Therefore, any non-zero-mean complex RV can always be generalized by a complex RV with a positive real mean from a statistical perspective. The distribution of the modulus of such a complex RV follows the Ricean distribution [11] ${'}_{R_{y}}=\frac{r_{y}}{\sigma_{y}^{2}}\exp\left(-\frac{r_{y}^{2}+{\left|u_{y}\right|}^{2}}{2\sigma_{y}^{2}}\right)I_{0}\left(\frac{r_{y}\left|\mu_{y}\right|}{\sigma_{y}^{2}}\right)$ where *I*_0_ is the modified Bessel function of the first kind with order zero. These distribution functions are used for *f_R__x_* and *f_R__y_* at the beginning of the derivation in Eq. (7).

## References

[1] Schoenecker S, Luginbuhl T (2016) Characteristic functions of the product of two Gaussian random variables and the product of a Gaussian and a gamma random variable. IEEE Signal Processing Letters 23(5):644-647.

[2] Astely D, Ottersten B (1999) The effects of local scattering on direction of arrival estimation with music. IEEE Transactions on Signal Processing 47(12):3220-3234.

[3] Baxley RJ, Walkenhorst BT, Acosta-Marum G (2010) Complex Gaussian ratio distribution with applications for error rate calculation in fading channels with imperfect CSI. 2010 IEEE Global Telecommunications Conference GLOBECOM 2010 (IEEE, Miami, FL, USA), pp 1-5.

[4] Wu S, Hughes BL (2018) Training-based joint channel and antenna impedance estimation. 52nd Annual Conference on Information Sciences and Systems (CISS) (IEEE, Princeton, NJ, USA), pp 1-6.

[5] Wu S (2019) Moments of complex Gaussian ratios. IEEE Communications Letters 23(1):88-91.

[6] Nadimi ES, Ramezani MH, Blanes-Vidal V (2018) On the ratio of independent complex Gaussian random variables. Multidimensional Systems and Signal Processing 29(4):1553-1561.

[7] Nadarajah S, Kwong H (2018) A note on "on the ratio of independent complex Gaussian random variables". Multidimensional Systems and Signal Processing 29(4):1839-1843.

[8] Li Y, He Q (2019) On the ratio of two correlated complex Gaussian random variables. IEEE Communications Letters 23(12):2172-2176.

[9] Gu D, Jargon J, Ryan M, Hubrechsen A (2020) Influence of noise on scattering-parameter measurements. IEEE Transactions on Microwave Theory and Techniques 10.1109/tmtt.2020.3014627 PMC8751672 35023878

[10] Papoulis A, Pillai SU (2002) Probability, Random Variables and Stochastic Processes (McGraw-Hill, London) 4th Ed.

[11] Rice SO (1944) Mathematical analysis of random noise. The Bell System Technical Journal 23(3):282-332.

[12] *NIST Digital Library of Mathematical Functions*. Release 1.0.24 of 2019-09-15. Olver FWJ, Olde Daalhuis AB, Lozier DW, Schneider BI, Boisvert RF, Clark CW, Miller BR, Saunders BV, Cohl HS, and McClain MA, eds. Available at https://dlmf.nist.gov/

[13] Watson GN (1995) Theory of Bessel Functions (Cambridge University Press, London) 2nd Ed. pp 383-449.

[14] Hyder MM, Khan RH, Mahata K (2014) An enhanced random access mechanism for smart grid M2M communications in WiMAX networks. 2014 IEEE International Conference on Smart Grid Communications (SmartGridComm) (IEEE, Venice, Italy), pp 356-361.

[15] Krishnamoorthy K, Mathew T (2009) Statistical Tolerance Regions: Theory, Applications, and Computation (Wiley, New York) 1st Ed. pp 25-53.

[16] *NIST/SEMATECH e-Handbook of Statistical Methods*. Last updated: 10/30/2013. Available at https://www.itl.nist.gov/div898/handbook/

[17] Forsythe GE, Malcolm MA, Moler CB (1976) Computer Methods for Mathematical Computations (Prentice Hall, Englewood Cliffs, New Jersey) 1st Ed. pp 122-133.

[18] Ng EW, Geller M (1969) A table of integrals of the error functions. Journal of Research of the National Institute Bureau of Standards-B Mathematical Sciences 73B(1):1-20.

